# Gut microbiome functional pathways outperform taxonomic profiles in predicting immune checkpoint inhibitor response in non-small cell lung cancer: an interpretable machine learning approach with SHAP

**DOI:** 10.3389/fimmu.2026.1832317

**Published:** 2026-05-15

**Authors:** Feifei Wei, Yoshiro Nakahara, Junya Isobe, Yuka Igarashi, Haruhiro Saito, Shuji Murakami, Tetsuro Kondo, Hidetomo Himuro, Taku Kouro, Tomoya Matsui, Satoshi Wada, Takuya Tsunoda, Kiyoshi Yoshimura, Tetsuro Sasada

**Affiliations:** 1 Division of Cancer Immunotherapy, Kanagawa Cancer Center Research Institute, Yokohama, Japan; 2 Cancer Vaccine and Immunotherapy Center, Kanagawa Cancer Center, Yokohama, Japan; 3 Department of Thoracic Oncology, Kanagawa Cancer Center, Yokohama, Japan; 4 Department of Respiratory Medicine, Kitasato University School of Medicine, Sagamihara, Japan; 5 Department of Hospital Pharmaceutics, School of Pharmacy, Showa Medical University, Tokyo, Japan; 6 Department of Obstetrics and Gynecology, Keio University School of Medicine, Tokyo, Japan; 7 Department of Clinical Diagnostic Oncology, Clinical Research Institute for Clinical Pharmacology and Therapeutics, Showa Medical University, Tokyo, Japan; 8 Division of Medical Oncology, Department of Medicine, School of Medicine, Showa Medical University, Tokyo, Japan; 9 Department of Clinical Immuno Oncology, Clinical Research Institute for Clinical Pharmacology and Therapeutics, Showa Medical University, Tokyo, Japan

**Keywords:** gut microbiome, immune checkpoint inhibitor, machine learning, metabolic pathway, non-small cell lung cancer, SHAP, treatment response

## Abstract

**Introduction:**

Lung cancer remains the leading cause of cancer mortality worldwide, with non-small cell lung cancer (NSCLC) accounting for the majority of cases. Although immune checkpoint inhibitors (ICIs) have transformed the therapeutic landscape of NSCLC, clinical responses remain highly variable. Emerging evidence implicates the gut microbiome in modulating the outcomes of ICI treatment; however, most studies to date have focused on taxonomic composition rather than microbial functional capacity. This study aimed to systematically compare the predictive value of taxonomic versus functional gut microbiome features across multiple ICI-related outcomes.

**Methods:**

Pretreatment fecal samples from 77 Japanese patients with NSCLC receiving ICIs were profiled using 16S rRNA sequencing. Six feature sets, comprising three taxonomic (family, genus, and species) and three functional (KEGG Orthology, Enzyme Commission, and MetaCyc pathways), were assessed using permutational multivariate analysis of variance for their association with clinical outcomes, including treatment response, irAEs, progression-free survival, and overall survival. Machine-learning models were subsequently developed based on MetaCyc pathway features to predict treatment response, with nested internal and external validation to ensure robustness and SHapley Additive exPlanations (SHAP) analysis for model interpretability.

**Results:**

Of all the feature sets tested, the functional profiles derived from the MetaCyc pathways exhibited the strongest association with the RECIST-defined response. A four-pathway signature, comprising PWY-4984 (urea cycle), SALVADEHYPOX-PWY (adenosine nucleotide degradation), OANTIGEN-PWY (O-antigen biosynthesis in *E. coli*), and PWY-5088 (L-glutamate degradation VIII to propanoate), achieved robust predictive performance, substantially outperforming any single feature. SHAP analysis confirmed that the primary drivers of responder classification were pathways involved in nitrogen metabolism and short-chain fatty acid biosynthesis.

**Conclusions:**

In this study, gut microbial functional profiles consistently outperformed taxonomic features in predicting ICI response in patients with NSCLC. These findings suggest that metabolic pathway-based signatures may capture functional microbiome-host interactions more effectively and hold greater promise as translatable, safer targets for precision intervention, particularly through metabolite-oriented strategies.

## 1 Introduction

Lung cancer remains the leading cause of cancer incidence and mortality worldwide, with non-small cell lung cancer (NSCLC) accounting for approximately 85% of all cases (1). A defining yet challenging aspect of immunotherapy, including immune checkpoint inhibitors (ICIs), is selective efficacy, with several studies identifying the gut microbiome as a potential biomarker for predicting treatment outcomes in patients with NSCLC (2–6). Previous studies have investigated the association between the structural features of the gut microbiome, such as alpha diversity, and the efficacy of ICI therapy. Overall, these reports consistently indicate that higher baseline microbial diversity is correlated with enhanced clinical response (7–9). Many studies have sought to correlate the taxonomic composition of the gut microbiome with the treatment outcomes of patients with NSCLC (6, 10–12). Although taxonomic features have substantial translational and modifiable potential, they also exhibit considerable diversity and complexity owing to differences in ethnicity, geographic environment, and dietary habits. Moreover, conclusions often vary depending on the taxonomic resolution applied, making it difficult to achieve broad consistency across different studies (5, 13–16). The gut microbiome is recognized as a “functional organ” that influences host physiology by modulating gene expression, thereby exerting its biological functions. Therefore, focusing on the characteristics of metabolic pathways and their products in this process is often more conducive to elucidating underlying mechanisms, which highlights the potential of functional features as more direct and precise biomarkers (14). Research into collective microbial behaviors, such as additive and synergistic effects and functional redundancy, has shown that microbial communities often converge on highly similar functional profiles despite variations in taxonomic composition (17, 18). Previous studies have shown that alterations in gut microbial functions are more predictive of host physiology and therapeutic responses than changes in specific microbial species (19–21).

A comprehensive evaluation was performed to compare the predictive performance of baseline gut microbiome features for treatment outcomes in 77 Japanese patients with NSCLC receiving ICIs. Using 16S rRNA sequencing data of pretreatment fecal samples, we generated taxonomic and functional profiles to systematically assess the associations with multiple clinical outcomes, including imaging-based response, immune-related adverse events (irAEs), progression-free survival (PFS), and overall survival (OS). To further explore the predictive capacity, we constructed machine learning models using the strongest target-predictor associations and interpreted the nonlinear random forest models via SHapley Additive exPlanations (SHAP) analysis. Originally proposed by Lloyd Shapley in 1953, SHAP applies game theory principles to quantify each participant’s contribution in a cooperative game (22), and is widely used for *post hoc* interpretation of black-box machine learning models (23–25). Our findings offer insights into the upper predictive limits of baseline gut microbiome data in this cohort, highlighting optimal data representations for specific clinical endpoints.

## 2 Materials and methods

### 2.1 Study design and participants

Seventy-seven patients with NSCLC who underwent treatment with anti-PD-1 (nivolumab or pembrolizumab) or anti-PD-L1 (atezolizumab) agents, administered either alone or in combination with chemotherapy, at Kanagawa Cancer Center (Yokohama, Japan) between May 2017 and January 2020 were included in this study. This cohort partially overlapped with a population previously reported by our group (26). Patient follow-up continued through July 2024, and baseline demographic and clinical characteristics are presented in **Table 1**. PD-L1 expression in tumor tissues was determined by immunohistochemical analysis of formalin-fixed paraffin-embedded sections using monoclonal antibodies against PD-L1 (clone E1L3N, Cell Signaling Technology, Danvers, MA, USA; and clone 22C3, Agilent Technologies/Dako, Carpinteria, CA, USA). For most patients, assessment was performed on specimens obtained before initiation of first-line therapy; the same procedure was applied to individuals treated with ICIs in later-line settings. Treatment response was evaluated based on the Response Evaluation Criteria in Solid Tumors (RECIST) version 1.1, while irAEs were graded using CTCAE version 5.0. The protocol complied with the Declaration of Helsinki and received approval from the Institutional Review Board of Kanagawa Cancer Center (approval no. 28-85). All participants provided written informed consent after receiving a detailed explanation of the study objectives and potential risks.

**Table 1:** Summary of patient characteristics in the study.

Patient characteristics	Total cohort
	(n = 77)
Age (years), mean (SD)	69.2 (8.1)
Sex, n (%)	
Female	16 (20.8)
Male	61 (79.2)
BMI, mean (SD)	22.1 (3.3)
Smoking, n (%)	
Former	64 (83.1)
Never	13 (16.9)
Stages, n (%)	
Stage III	7 (9.1)
Stage IV	45 (58.4)
Recurrence	25 (32.5)
Histology, n (%)	
Non-squamous	56 (72.7)
Squamous	21 (27.3)
Driver mutation, n (%)	
Wild type	65 (84.4)
EGFR	6 (7.8)
ALK	0 (0)
Unknown	6 (7.8)
Tumor PD-L1 expression, n (%)	
<1%	14 (18.2)
1%-49%	17 (22.1)
≥ 50%	39 (50.6)
Unknown	7 (9.1)
Treatment line, n (%)	
1st line	29 (37.7)
Further lines	48 (62.3)
Prior therapy, n (%)	
Chemotherapy	37 (48.1)
Chemotherapy and surgery	3 (3.9)
Chemotherapy and radiation	7 (9.1)
Radiation	3 (3.9)
None	27 (35.1)
ECOG PS, n (%)	
0	32 (41.6)
1	36 (46.8)
2	9 (11.7)
Antibiotics, n (%) ^1^	
Yes	33 (42.9)
No	44 (57.1)
Proton-pump inhibitors, n (%) ^2^	
Yes	35 (45.5)
No	42 (54.5)
Corticosteroids, n (%) ^2^	
Yes	4 (5.2)
No	73 (94.8)
Treatment option, n (%)	
Monotherapy	33 (42.9)
Combination therapy	44 (57.1)
Anti-PD-1 or anti-PD-L1 antibodies, n (%)	
Anti-PD-1	64 (83.1)
Anti-PD-L1	13 (16.9)
Occurrence of irAEs, n (%)	
Yes	30 (39.0)
No	47 (61.0)
Best clinical response (RECIST), n (%)	
Partial response	28 (36.4)
Stable disease	13 (16.9)
Progressive disease	33 (42.9)
Unknown	3 (3.9)
Progression-free survival (days), median (95% Cl) ^3^	148 (117-325)
Overall survival (days), median (95% CI) ^3^	653 (347-1039)

ALK, anaplastic lymphoma kinase; BMI, body mass index; CI, confidence interval; ECOG PS, Eastern Cooperative Oncology Group performance status; EGFR, epidermal growth factor receptor; irAE, immune-related adverse event; RECIST, Response Evaluation Criteria in Solid Tumors; SD, standard deviation. ^1^ “Yes” indicates use within the three months preceding treatment initiation. ^2^ “Yes” indicates use initiated before and ongoing at treatment initiation. ^3^ Kaplan–Meier method

### 2.2 Sample collection

Fecal samples were collected within two weeks prior to therapy initiation using a stool collection kit containing guanidine (TechnoSuruga Laboratory, Shizuoka, Japan) and immediately stored at -80 °C until analysis.

### 2.3 DNA extraction, gene amplification, sequencing, and data analysis

DNA was extracted using the QIAamp PowerFecal Pro DNA Kit (QIAGEN, Hilden, Germany) in accordance with the manufacturer’s protocol. The V3-V4 regions of the 16S rRNA gene were amplified and sequenced on an Illumina MiSeq platform (Illumina, San Diego, CA, USA) using paired-end reads (2 × 300 bp). Raw reads were processed using QIIME2 (version 2024.10; https://qiime2.org/) . Demultiplexed paired-end sequences were imported in Casava 1.8 format and denoised using the DADA2 plugin, including quality filtering, error correction, paired-end merging, and chimera removal. The first 20 bases of both forward and reverse reads were trimmed, and reads were truncated at 250 bp, resulting in amplicon sequence variants (ASVs) (27). Taxonomic classification was performed using a Naïve Bayes classifier trained on the SILVA 138 database (99% identity, trimmed to the V3-V4 region). ASVs assigned to mitochondria or chloroplasts were removed prior to downstream analysis, and taxonomic composition was summarized using the q2-taxa plugin. For functional prediction, ASV sequences and their corresponding abundance table were exported from QIIME2 and used as input for PICRUSt2 (version 2.6.0; https://github.com/picrust/picrust2) via the standalone pipeline (28). Gene family abundances were inferred through sequence placement using EPA-NG followed by hidden-state prediction, and normalized by predicted 16S rRNA gene copy number. Functional profiles were generated based on Enzyme Commission (EC), KEGG Orthology (KO), and MetaCyc databases, with pathway inference using MinPath.

### 2.4 Machine learning and statistical analysis

All analyses were conducted using the R software (version 4.4.1; https://www.r-project.org). Bray–Curtis distances were calculated using the vegdist function, and permutational multivariate analysis of variance (PERMANOVA) was performed using the adonis2 function in the vegan package. Univariate binary logistic regression analyses were performed using the glm function in R basic package, and P-values were derived using Wald tests and subsequently adjusted for multiple comparisons using the Benjamini–Hochberg false discovery rate (FDR) procedure.

For machine learning analyses, the cohort was randomly stratified into training (80%) and test (20%) sets. Model development and hyperparameter optimization were conducted using nested cross-validation (CV) exclusively within the training set. Random forest (RF) modeling was implemented as described in our previous studies (26, 29, 30). Feature selection was performed via inclusion of sequential variables based on importance rankings, with the variable importance assessed using the mean Gini index. The optimal feature subset was determined by maximizing the predictive accuracy during the addition of the stepwise variable. Model performance was assessed using leave-one-out cross-validation (LOO-CV) of the training set with discriminatory power quantified by the area under the receiver operating characteristic curve (AUC). The final models were subsequently retrained on the entire training set and independently evaluated on the held-out test set, maintaining a strict separation between the development and evaluation datasets to prevent data leakage. All RF models consisted of 1,000 decorrelated decision trees with the minimum node size set to 1, in line with established practices for high-dimensional data. Permutation testing was conducted by randomly shuffling the outcome labels in the test set 1,000 times using the sample function to generate empirical null distributions. For SHAP interpretation, the explain function in the fastshap package was applied to obtain SHAP values for each sample when it served as the test instance in the LOO framework. A higher mean absolute SHAP value corresponds to a greater average magnitude of influence, regardless of direction, that a feature exerts on the model’s predictions. Spearman correlation coefficients and corresponding P-values were calculated using the rcorr function in the hmisc package. Nonparametric comparisons were performed using the Wilcoxon signed-rank test via the wilcox.test function. The survival analyses included Kaplan–Meier estimations implemented in the survival packages. The analysis code used in this study is available in the **Supplementary Materials**.

## 3 Results

### 3.1 Patient characteristics

Patient characteristics are summarized in **Table 1**. The cohort consisted of 77 patients with a mean age of 69.2 years; 79.2% were male and 20.8% were female. The mean body mass index (BMI) was 22.1 kg/m^2^, and 83.1% of patients had a history of smoking. Regarding disease stage, 9.1% had stage III disease, 58.4% had stage IV disease, and 32.5% presented with recurrence. Histologically, 72.7% had non-squamous carcinoma. Driver mutation testing identified EGFR mutations in six patients, whereas no ALK rearrangements were detected, yielding an overall detection rate of 7.8%. PD-L1 expression ≥ 50% was observed in 39 patients (50.6%), 1-49% in 17 patients (22.1%), and < 1% in 14 patients (18.2%); PD-L1 status was unavailable in seven patients (9.1%) due to the lack of adequate tumor tissue. First-line ICI therapy was administered to 37.7% of patients, while the remainder received ICIs as further-line treatment. Prior treatment histories included chemotherapy alone in 37 patients, chemotherapy plus surgery in three patients, chemotherapy plus radiotherapy in seven patients, and radiotherapy alone in three patients. The distribution of ECOG performance status (PS) was as follows: PS 0 in 41.6%, PS 1 in 46.8%, and PS 2 in 11.7% of patients. Regarding treatment regimens, 42.9% and 57.1% received ICI monotherapy and combination therapy, respectively. Regarding treatment outcomes, irAEs occurred in 39.0% of patients; based on RECIST evaluation, 28 (36.4%) patients achieved partial response (PR), 13 (16.9%) had stable disease (SD), and 33 (42.9%) showed progressive disease (PD), and RECIST assessment was unavailable in three patients; the median PFS for the entire cohort was 148 days, and the median OS was 653 days.

### 3.2 Functional pathway-based descriptors exhibit stronger correlations with immune checkpoint inhibitor response than taxonomic profiles

**Figure 1:**
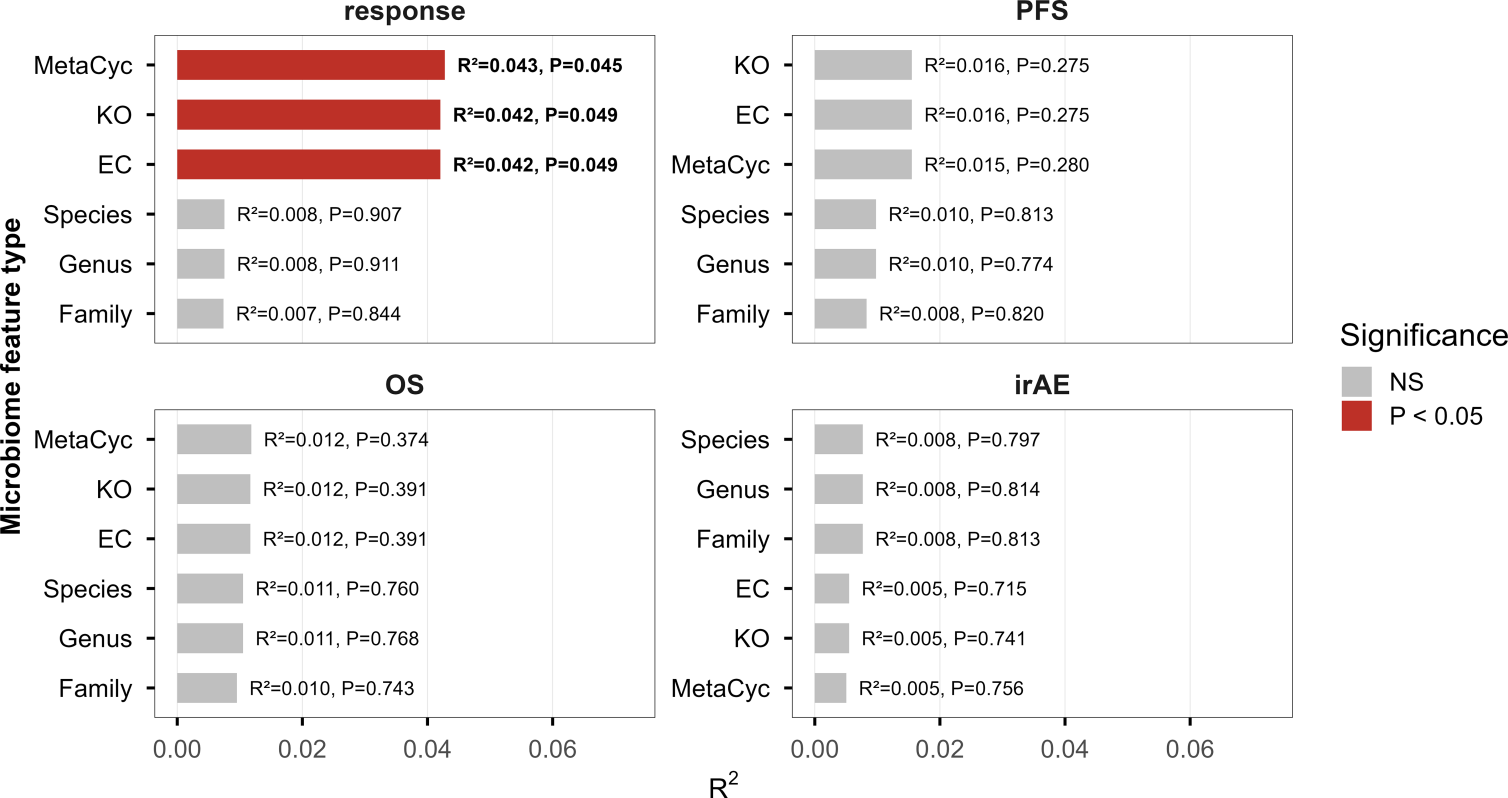
Explanatory power of Bray–Curtis distance matrices derived from taxonomic and functional gut microbiome features for clinical outcomes of ICI treatment assessed using PERMANOVA (N = 77). The red bars indicate features for which PERMANOVA identified a statistically significant association with the outcome (P < 0.05). Abbreviations: EC, Enzyme Commission-based features; ICI, immune checkpoint inhibitor; KO, KEGG Orthology-based features; MetaCyc, MetaCyc pathway-based features; NS, not significant; OS, overall survival; P, P value; PERMANOVA, permutational multivariate analysis of variance; PFS, progression-free survival; RECIST, Response Evaluation Criteria in Solid Tumors.

To assess the associations between gut microbiome feature types (taxonomic and functional) and each treatment outcome, Bray-Curtis distances were firstly calculated for each feature matrix, and PERMANOVA was applied to quantify the explanatory power of these distance matrices with respect to the corresponding outcomes (**Figure 1**). As shown in **Figure 1**, distance matrices derived from functional features mapped to the MetaCyc, KO, and EC databases showed significant associations with RECIST response in PERMANOVA tests. Among these, MetaCyc pathway-based features exhibited the strongest association. In contrast, taxonomic features demonstrated markedly weaker explanatory power for RECIST outcomes compared to all functional datasets. While neither functional nor taxonomic features reached statistical significance for PFS or OS, functional matrices consistently accounted for a larger proportion of variance. Conversely, for irAEs, taxonomic feature matrices displayed greater explanatory power than their functional counterparts. These results indicate that the therapeutic efficacy of ICIs may be more strongly linked to the collective functional capacity of the gut microbiome, especially at the pathway level, whereas the occurrence of irAEs might be more closely tied to the presence or abundance of specific microbial taxa.

### 3.3 Univariate logistic regression identifies baseline gut microbial metabolic pathways that are significantly associated with immune checkpoint inhibitor response

To explore the gut microbiome’s predictive limit for treatment outcomes in this cohort, we focused on MetaCyc pathway features as predictors of RECIST-based response.

Before multivariate modeling, univariate logistic regression was performed on each of the 516 successfully mapped MetaCyc pathways across the 77 samples to evaluate their associations with the RECIST response. A total of 195 pathways showed nominal significance (P < 0.05), although none remained significant after FDR correction (**Supplementary Table 1**). Five pathways were identified with P < 0.01: PWY-7389 (superpathway of anaerobic energy metabolism [invertebrates]) and PWY-7383 (anaerobic energy metabolism [invertebrates, cytosol]), CITRULBIO-PWY (L-citrulline biosynthesis), PWY-6922 (L-N^δ^-acetylornithine biosynthesis), and PWY-4984 (urea cycle). When assessed individually for their ability to discriminate treatment response, PWY-7389 achieved the highest predictive performance with an AUC of 0.683. Notably, the most significant pathways were primarily related to microbial nitrogen and energy metabolism.

### 3.4 Machine learning-based high-dimensional data analysis framework reveals a metabolic pathway signature that predicts immune checkpoint inhibitor response with high accuracy and reproducibility

To identify multivariate feature patterns associated with treatment response, we subsequently established a machine learning-based biomarker discovery framework derived from functional features of gut microbiome.

**Figure 2:**
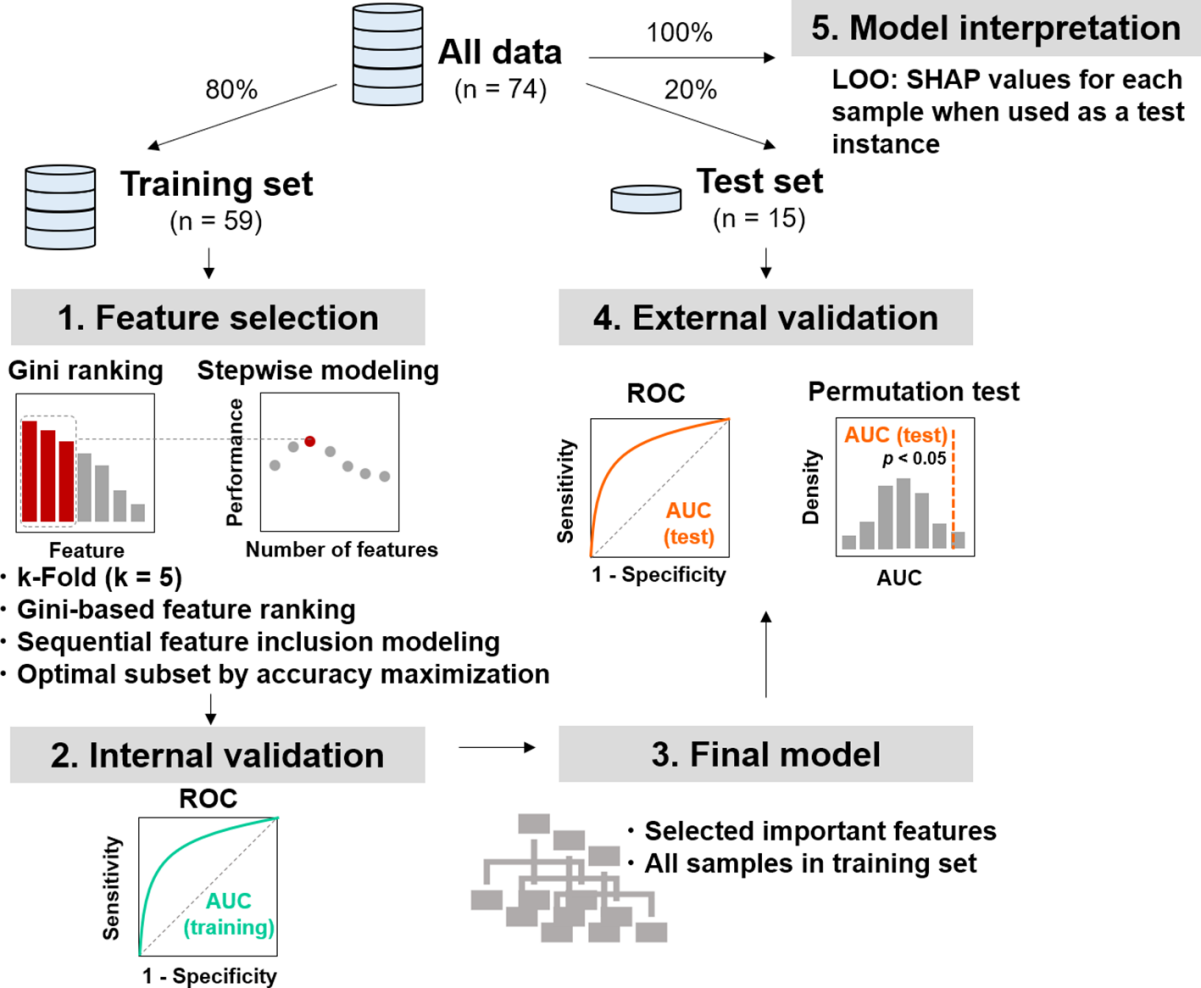
Machine learning pipeline for predicting ICI response based on MetaCyc functional pathway features (N = 74). A total of 74 patients with RECIST-defined ICI response labels were included in the analysis. The dataset was randomly partitioned into a training set (80%, N = 59) and an independent external test set (20%, N = 15). The analytical workflow comprised five sequential steps. (1) Feature selection: Within the training set, a random forest classifier was trained using k-fold cross-validation (k = 5). Candidate features were first ranked by Gini importance, followed by stepwise sequential feature inclusion modeling, in which features were added in descending order of importance one at a time. The optimal feature subset was defined as the combination that yielded the highest predictive accuracy. (2) Internal validation: Model performance was assessed within the training set using the AUC, with the ROC curve illustrating discrimination between responders and non-responders. (3) Construction of the final model: The final model was trained on all samples in the training set using the selected optimal feature subset. (4) External validation: The final model was applied to the independent test set. Discrimination performance was quantified by AUC on the test set and further evaluated by permutation testing, in which the observed test AUC was compared against a null distribution generated by randomly permuting outcome labels. (5) Model interpretation: to assess feature contributions at the individual sample level, an LOO strategy was employed in conjunction with SHAP analysis, whereby each sample in the full dataset was iteratively used as a test instance to derive its corresponding SHAP values, enabling visualization of the direction and magnitude of each feature’s contribution to model predictions. Abbreviations: AUC, area under the receiver operating characteristic curve; ICI, immune checkpoint inhibitor; LOO, leave-one-out; ROC receiver operating characteristic curve; SHAP, SHapley Additive exPlanations.

**Figure 3:**
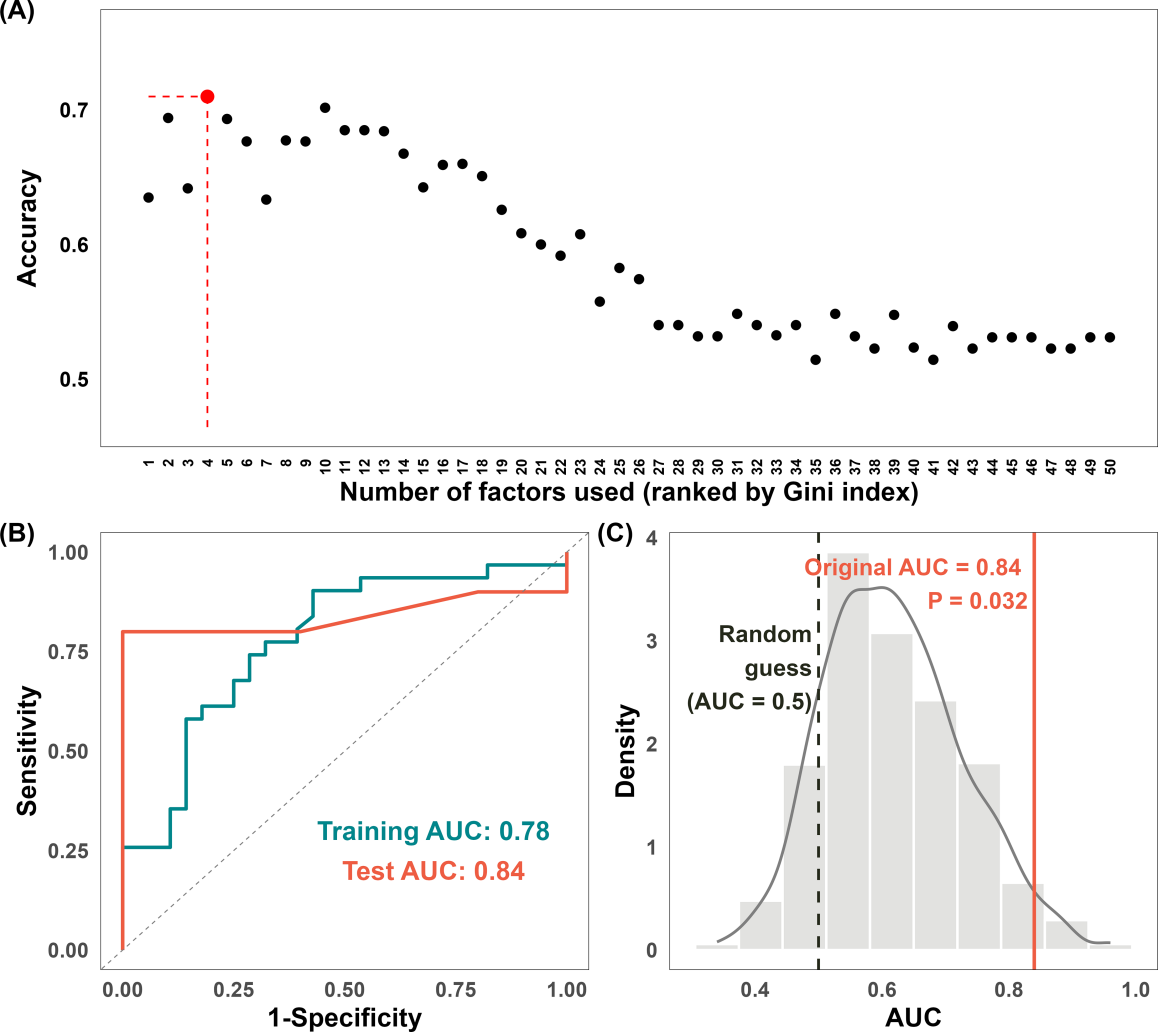
Predictive performance of the random forest model for ICI response classification based on MetaCyc functional pathway features (N = 74). **(A)** Model accuracy as a function of the number of features included. The optimal feature subset was identified at four features, corresponding to the point of maximum predictive accuracy. **(B)** The corresponding ROC curves of training and test sets, illustrating the discriminative performance of the final model. **(C)** AUC distribution derived from permutation testing on the test set (1,000 permutations), demonstrating that the observed test AUC significantly exceeds the null distribution (p < 0.05). Abbreviations: AUC, area under the receiver operating characteristic curve; ICI, immune checkpoint inhibitor; P, P value; ROC, receiver operating characteristic curve.

Patients with available RECIST labels (n = 74) were randomly divided into training (80%) and independent test (20%) sets (**Figure 2**). Feature selection and hyperparameter tuning were performed on the training set. Following internal validation, a final model was trained on the full training set and evaluated on the held−out test set for external validation. Model performance was quantified using the AUC. Permutation testing was performed to confirm that the observed performance was not due to chance. Throughout this process, strict safeguards, including the use of CV within the training phase and a completely separate test set, were enforced to prevent data leakage and control overfitting. A sequential feature selection procedure was embedded in the workflow prior to model construction using the training set. An initial model was built using all features, and the importance of the variables was assessed. Then, features were sequentially incorporated into the model in descending order of importance, with predictive performance recorded at each step. As shown in **Figure 3A**, the model incorporating the four most important features achieved the highest accuracy, whereas the inclusion of additional features increased structural noise and reduced model performance. These four selected features were subsequently used for LOO-CV within the training set. A model was trained on all remaining training samples for each target sample to maximize data use and generate fold-independent predictions. The corresponding ROC curves are shown in **Figure 3B**, with AUCs of 0.78 in the training, and 0.84 in the test sets, respectively. Permutation testing on the test set was conducted to assess the model’s validity. Random shuffling of the response labels yielded a distribution of AUC values centered at 0.5 (gray density plot**;** **Figure 3C**), indicating chance-level performance. This distribution was significantly lower than the true-label AUC of 0.84 (green line; P = 0.032). These results demonstrate that a combination of gut microbiome metabolic functional features can predict ICI treatment response with high accuracy and that the model’s predictive performance is robust and nonrandom to an independent test set.

To examine the potential influence of clinical heterogeneity on model performance, we constructed two additional predictive models: one based exclusively on clinical variables, and the other integrating both clinical and microbiome features. As shown in **Supplementary Figures 1A**-**1C**, using the identical training-test split and machine learning pipeline, the clinical-only model identified three important predictive variables, including age, BMI, and prior therapy, but failed to demonstrate meaningful predictive performance in either the training or test set, with AUC values of 0.51 and 0.50, respectively. As shown in **Supplementary Figures 1D, F**, when clinical and selected microbiome features were combined, the machine learning pipeline identified two important variables, namely OANTIGEN-PWY and PWY-5088, and the resulting model exhibited a substantial improvement in predictive performance relative to the clinical-only model, with AUC values of 0.72 and 0.70 for the training and test sets, respectively. Nevertheless, the performance of the combined model did not exceed that of the model trained exclusively on microbiome functional features (**Figure 3B**), with AUC values of 0.78 and 0.84 for the training and test sets, respectively. Collectively, these findings suggest that the clinical variables examined in this cohort provided limited predictive value for ICI response, and their inclusion alongside microbiome functional features did not improve upon the microbiome-only model. This pattern may indicate that clinical heterogeneity introduced noise that partially attenuated the predictive signal derived from microbial functional features, further supporting the potential of gut microbiome functional profiling as an independent source of predictive information.

Given the growing clinical interest in risk stratification for ICI-related toxicity, we performed an additional machine learning analysis using the species-level gut microbial features most strongly associated with irAE status, as identified in **Figure 1**. The optimal model incorporated four features but yielded modest discriminative performance, with AUC values of 0.61 and 0.58 in the training and test sets, respectively. Permutation testing on the test set revealed no significant difference between the observed test AUC and the null distribution (P = 0.59), indicating that the gut microbial features evaluated in this study were insufficient to predict irAE occurrence beyond chance in this cohort (**Supplementary Figure 2**).

### 3.5 SHapley Additive exPlanations analysis elucidates nonlinear feature characteristics associated with immune checkpoint inhibitor response

**Figure 4:**
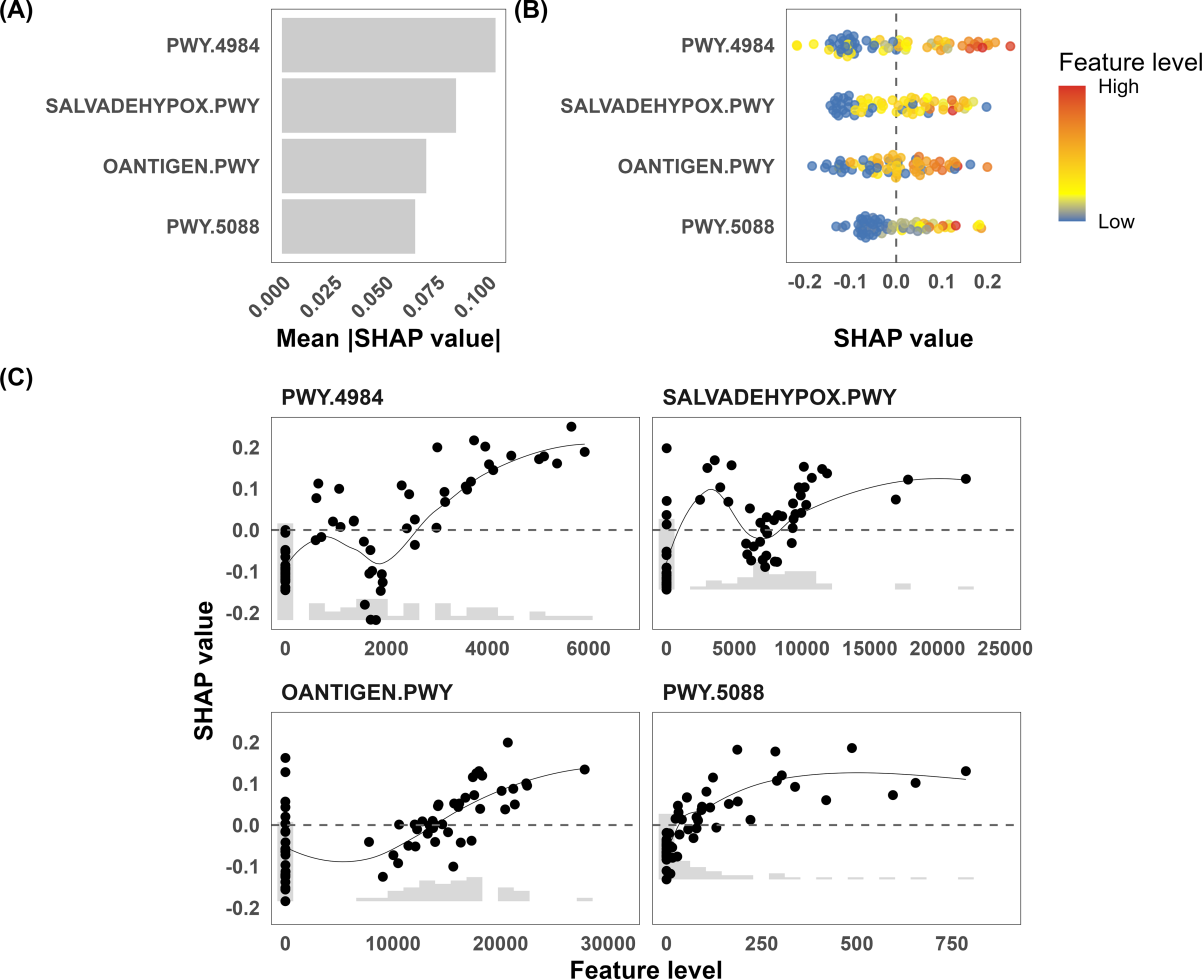
SHAP analysis of the functional pathways associated with the ICI response (N = 74). **(A)** Mean absolute SHAP values for the four selected MetaCyc pathways: PWY-4984 (urea cycle), SALVADEHYPOX-PWY (adenosine nucleotide degradation), OANTIGEN-PWY (O-antigen building blocks biosynthesis in Escherichia coli), and PWY-5088 (L-glutamate degradation VIII to propanoate), reflecting the relative contribution of each pathway to model predictions. **(B)** Beeswarm plot illustrating the distribution of SHAP values across individual samples. **(C)** Dependence plots showing the relationship between feature abundance and SHAP value for each of the four selected pathways. Abbreviations: ICI, immune checkpoint inhibitor; P, P value; SHAP, SHapley Additive exPlanations.

To improve the interpretability of the nonlinear random forest model, we applied SHAP analysis using test predictions from LOO-CV to avoid data leakage (**Figure 2**). The mean absolute SHAP values identified PWY-4984 (urea cycle) as the most influential feature, followed by SALVADEHYPOX-PWY (adenosine nucleotide degradation), OANTIGEN-PWY (O-antigen building blocks biosynthesis *[E. coli]*), and PWY-5088 (L-glutamate degradation VIII to propanoate) (**Figure 4A**). A beeswarm plot (**Figure 4B**) illustrates the direction of each feature’s influence on predictions, with positive and negative SHAP values indicating shifts toward response and non-response, respectively. PWY-5088 exhibited a near-monotonic relationship, with higher pathway activity promoting the response. In contrast, PWY-4984 and SALVADEHYPOX-PWY showed pronounced biphasic patterns, wherein SHAP values initially increased at low activity levels, declined at intermediate levels, and rose again at higher levels, indicating multiple activity ranges associated with the predicted response. OANTIGEN-PWY demonstrated a non-monotonic pattern, in which both low and intermediate levels were associated with non-response, whereas high levels favored response (**Figure 4C**). Notably, Spearman correlation analysis revealed a strong co-abundance relationship between PWY-4984 and SALVADEHYPOX-PWY (**Supplementary Figure 3**), which is consistent with their coordinated SHAP patterns and suggests that PWY-4984 and SALVADEHYPOX-PWY make joint contributions to model predictions.

## 4 Discussion

This study analyzed pretreatment gut microbiome 16S rRNA sequencing data from 77 Japanese patients with NSCLC who received ICI therapy. Using PERMANOVA, we evaluated the explanatory power of six microbiome feature datasets, comprising three taxonomic (family, genus, and species) and three functional (KO, EC, and MetaCyc pathway/enzyme profiles), for multiple clinical outcomes (treatment response, irAE, PFS, and OS). Among these, the MetaCyc pathway enrichment score demonstrated the strongest association with treatment response. Focusing on the MetaCyc functional pathway dataset, we subsequently performed machine learning modeling to predict the RECIST-based response. This approach identified a four-pathway signature, comprising PWY-4984 (urea cycle), SALVADEHYPOX-PWY (adenosine nucleotide degradation), OANTIGEN-PWY (O-antigen building blocks biosynthesis [*E. coli*]), and PWY-5088 (L-glutamate degradation VIII to propanoate), that generated a highly accurate and robust predictive model. To interpret the nonlinear model, SHAP analysis was employed to quantify and characterize the contribution of individual features. Overall, our results demonstrate that functional profiles of the gut microbiome outperform taxonomic profiles in predicting ICI response, highlighting that specific microbial metabolic activities may better capture the biological mechanisms underlying the interaction between the microbiome and cancer immunotherapy.

PWY-4984 corresponds to the microbial urea cycle, in which amino groups are converted to urea with L-arginine, L-ornithine, and L-citrulline as intermediates. This pathway showed the strongest association with ICI response in our cohort. Previous studies have linked gut bacterial nitrogen recycling to disease progression and therapeutic sensitivity in multiple myeloma (31, 32), and arginine metabolism, which serves as a key intermediate of this pathway, has been implicated as a determinant of antitumor immune activation (33–36). Emerging evidence further implicates the urea cycle in the metabolic reprogramming of the tumor immune microenvironment (37). Our findings raise the possibility that gut microbial urea cycle activity may be associated with immunomodulatory metabolite availability; however, these observations are hypothesis-generating and require experimental validation. SALVADEHYPOX-PWY corresponds to adenosine nucleotide degradation, which releases ammonia as a by-product of adenosine monophosphate catabolism. In contrast to the urea cycle, which assimilates ammonia, this pathway generates it, and the activities of the two pathways were highly correlated in our cohort. This suggests that the balance between microbial ammonia production and assimilation may jointly influence the ICI response, although the underlying mechanisms remain to be established.

OANTIGEN-PWY represents the O-antigen building block biosynthesis in gram-negative bacteria. The O-antigen is the most variable region of lipopolysaccharide and serves as a major B-cell antigen capable of eliciting antigen-specific immune responses (38, 39); microbial O-antigens have also been reported to promote natural killer cell activation (40). In this study, higher abundance of this pathway was positively associated with predicted ICI response, suggesting a possible link between O-antigen-related immune stimulation and ICI efficacy. PWY-5088 corresponds to L-glutamate degradation VIII to propanoate, through which gut microbes produce short-chain fatty acids (SCFAs) including propionate. Previous studies have reported associations between fecal SCFA levels and ICI response (41, 42), and mechanistic studies have suggested roles for SCFAs in modulating CD8^+^ T cell differentiation (43, 44). Propionate accounted for approximately 19% of the total fecal SCFA pool patients with NSCLC receiving ICI therapy (45). Consistent with these findings, we observed a monotonic positive association between this pathway and the predicted treatment response, which may warrant further investigation.

In our study, the gut microbial functional signature comprising these four metabolic pathways demonstrated superior predictive performance for treatment response compared with analyses based on any single feature. From a translational perspective, although taxon-based approaches offer more readily translatable targets, focusing on microbial functions provides distinct advantages. Strategies centered on microbial metabolites, rather than live organisms, can improve safety by avoiding the uncontrolled microbial proliferation or translocation risks. Indeed, metabolite-based interventions have shown efficacy comparable to live biotherapies, such as fecal microbiota transplantation, while offering greater controllability (46, 47).

This study has several limitations. First, the sample size is small relative to the feature dimensionality, which may introduce feature selection bias. To mitigate this concern, we implemented a structured analytical framework including internal k-fold CV, external validation on an independent test set, and permutation testing. Although these methodological safeguards reduce the risk of overfitting, the possibility of residual bias cannot be entirely excluded. Furthermore, the generalizability of the present findings requires prospective confirmation in larger, independent cohorts. Second, functional pathway inference was based on 16S rRNA gene sequencing rather than shotgun metagenomics; this approach has inherent limitations: the 16S marker gene provides limited phylogenetic resolution at the species and strain level, and the completeness and taxonomic coverage of reference databases limit the inferred functional profiles (48, 49). Third, the functional interpretations of the microbiome in this study were largely based on pathway inference and associative analyses; therefore, the proposed mechanisms remain at a hypothesis-generating stage. More reproducible and broadly applicable conclusions will require validation in larger, independent cohorts and rigorous experimental studies designed to test these hypotheses. As a pilot study, our work provides an initial perspective on the potential role of gut microbial metabolic functions in shaping responses to ICI therapy in NSCLC. Growing evidence confirms that the gut microbiome influences not only carcinogenesis but also cancer therapy efficacy and toxicity. Integrating microbial metabolic profiling and host-microbiome co-metabolism into therapeutic design represents an emerging paradigm in precision oncology.

## Data Availability

The datasets presented in this study can be found in online repositories. The names of the repository/repositories and accession number(s) can be found below: https://www.ncbi.nlm.nih.gov/bioproject/PRJNA1438385.
