# Supplementary material for: Gut microbiome functional pathways outperform taxonomic profiles in predicting immune checkpoint inhibitor response in non-small cell lung cancer: an interpretable machine learning approach with SHAP
Source: Front Immunol. 2026 May 15;17:1832317. doi: 10.3389/fimmu.2026.1832317 (PMC13218901; doi:10.3389/fimmu.2026.1832317)
Supplement: Supplementary file 2 [file DataSheet2.pdf]

```r
###########################################################
# Title:
# Analysis Code for:
# "Gut Microbiome Functional Pathways Outperform Taxonomic
# Profiles in Predicting Immune Checkpoint Inhibitor Response
# in Non–Small Cell Lung Cancer: An Interpretable Machine
# Learning Approach with SHAP"
#
# Correspondence: Feifei Wei (amweifeifei@gmail.com)
#
# Description:
# This script contains the main analysis pipeline used in the study,
# including data preprocessing, feature selection, model training,
# evaluation, and SHAP-based interpretation.
#
# Notes:
# - Raw data are not included in this repository.
# - Please ensure all required packages are installed before running.
# - Code was developed and tested in R (version 4.4.1).
###########################################################

# ---- Load required packages ----
library(tidyverse)
library(survival)
library(survminer)
library(survivalROC)
library(survAUC)
library(timeROC)
library(ggrepel)
library(pROC)
library(caret)
library(rlist)
library(rstatix)
library(ggpubr)
library(PRROC)
library(Rtsne)
library(gridExtra)
library(igraph)
library(randomForestSRC)
library(randomForest)
library(heatmaply)
library(EnhancedVolcano)
```

```r
library(grid)
library(glmnet)
library(forestplot)
library(vegan)
library(ggpmisc)
library(yardstick)
library(plotly)
library(mixOmics)
library(KEGGREST)
library(patchwork)
library(matrixStats)
library(wesanderson)
library(UpSetR)
library(ComplexUpset)
library(fastshap)
library(future)
library(parallel)
library(tidytext)
library(mlr)

# Set seed for reproducibility
seed <- 123

############################################################
# PERMANOVA
############################################################
# x: microbiome datasets (x1:species; x2:genus; x3:family; x4:KO; x5:EC, x6:MetaCyc)
# y: clinical outcomes (y1:RECIST-based response; y2:PFS; y3:OS; y4:irAE)
y1 <- y1 %>%
  mutate(
    response = case_when(
      RECIST=="PD"~ 0,
      RECIST=="SD"~ 1,
      RECIST=="PR"~ 1
      )
    ) %>%
  pull(response)

y2 <- y2 %>%
  mutate(
    pfs360 = case_when(
      PFS > 360 ~ 1,
```

```
      PFS == 360 & PFS_censor == 1 ~ 1,
      PFS == 360 & PFS_censor == 0 ~ 0,
      PFS < 360 & PFS_censor == 0 ~ 0,
      PFS < 360 & PFS_censor == 1 ~ NA
    )
  ) %>%
  pull(pfs360)

y3 <- y3 %>%
  mutate(
    os360 = case_when(
      OS > 360 ~ 1,
      OS == 360 & OS_censor == 1 ~ 1,
      OS == 360 & OS_censor == 0 ~ 0,
      OS < 360 & OS_censor == 0 ~ 0,
      OS < 360 & OS_censor == 1 ~ NA
    )
  ) %>%
  pull(os360)

x_list <- list(
  Species = x1,
  Genus   = x2,
  Family  = x3,
  KO      = x4,
  EC      = x5,
  MetaCyc = x6
)

y_list <- list(
  response = y1,
  PFS    = y2,
  OS     = y3,
  irAE   = y4
)

adonis_result <- list()

for (xname in names(x_list)) {
  for (yname in names(y_list)) {

    key <- paste(xname, yname, sep = "_")
```

```
    adonis_result[[key]] <- adonis2(
      vegdist(x_list[[xname]], method = "bray") ~ .,
      data = y_list[[yname]]
    )

    cat("Done:", key, "¥n")
  }
}

############################################################
# Machine learning: response ~ MetaCyc
############################################################

# ---- data preprocessing ----
df <- data.frame(y_list[["response"]], x_list[["MetaCyc"]])
colnames(df)[1] <- "y"
df$y <- as.factor(df$y)

set.seed(seed)
training_rownum <- sample(seq_len(nrow(df)), size = floor(0.8 * nrow(df)))
training <- df[training_rownum, ]
test     <- df[-training_rownum, ]

# ---- feature selection (Gini + CV-ACC) ----
kFold <- makeResampleDesc("RepCV", fold = 5, reps = 5)
RF    <- makeLearner(cl = "classif.randomForest", id = "rf", predict.type = "prob")
Task <- makeClassifTask(data = training, target = "y", positive = "1")

set.seed(seed)
res <- resample(
  learner    = RF,
  task       = Task,
  resampling = kFold,
  extract    = getFeatureImportance
)

n_iter <- kFold$folds * kFold$reps

imp_list <- lapply(seq_len(n_iter), function(i) {
  res$extract[[i]]$res$importance
})
```

```r
gini <- data.frame(
  name = colnames(training[, -1]),
  gini = rowSums(do.call(cbind, imp_list))
) %>%
  arrange(desc(gini))

acc_mat <- matrix(NA, nrow = nrow(gini), ncol = kFold$folds * kFold$reps)

for (i in seq_len(nrow(gini))) {

  feature_sel <- gini$name[1:i]
  dat_sub     <- training %>% dplyr::select(y, all_of(feature_sel))
  Task_sub    <- makeClassifTask(data = dat_sub, target = "y", positive = "1")


  res_sub <- resample(
    learner    = RF,
    task       = Task_sub,
    resampling = kFold,
    measures   = list(acc)
  )

  acc_mat[i, ] <- res_sub$measures.test$acc
}
feature_vip <- gini$name[1:which.max(rowMeans(acc_mat))]

# ---- model training & LOO-CV ----
dat_tr <- training %>% dplyr::select(y, any_of(feature_vip))
Task_tr <- makeClassifTask(data = dat_tr, target = "y", positive = "1")

set.seed(seed)
res_tr <- resample(
  learner    = RF,
  task       = Task_tr,
  resampling = makeResampleDesc("LOO")
)

prob_tr <- res_tr$pred$data$prob.1
ROC_tr  <- roc(y ~ prob_tr, data = data.frame(prob_tr, dat_tr), ci = TRUE)
AUC_tr  <- round(ROC_tr$auc, digits = 3)
cat("AUC (Training LOO):", AUC_tr, "¥n")
```

```
# ---- final model & test ----
dat_te <- test %>% dplyr::select(y, any_of(feature_vip))

final_model   <- mlr::train(learner = RF, task = Task_tr)
pred_te <- predict(final_model, newdata = dat_te)

prob_te <- pred_te$data$prob.1
ROC_te  <- roc(y ~ prob_te, data = data.frame(prob_te, dat_te), ci = TRUE)
AUC_te  <- round(ROC_te$auc, digits = 3)

cat("AUC (Test):", AUC_te, "¥n")

# ---- permutation test ----
set.seed(seed)
rf_fit <- randomForest(
  x     = dat_tr %>% dplyr::select(-y),
  y     = dat_tr$y,
  ntree = 1000,
  mtry  = floor(sqrt(ncol(dat_tr) - 1))
)

pred_prob <- predict(
  rf_fit,
  newdata = dat_te %>% dplyr::select(-y),
  type    = "prob"
)[, 2]

AUC_original <- auc(roc(response = dat_te$y, predictor = pred_prob))
cat("AUC (original):", round(AUC_original, 3), "¥n")

nperm    <- 1000
auc_perm <- numeric(nperm)

for (i in seq_len(nperm)) {
  y_perm     <- sample(dat_te$y)
  auc_perm[i] <- auc(roc(response = y_perm, predictor = pred_prob, quiet = TRUE))
}

p_value  <- (sum(auc_perm >= AUC_original) + 1) / (nperm + 1)
perm_df  <- data.frame(AUC = auc_perm)
```

```r
cat("P value (permutation):", round(p_value, 4), "¥n")


# ---- LOO-SHAP ----
df_shap <- df %>% dplyr::select(y, any_of(feature_vip))

X <- as.data.frame(df_shap %>% dplyr::select(-y))
y <- df_shap$y
n <- nrow(X)
p <- ncol(X)

pred_loo <- numeric(n)
shap_loo <- matrix(NA, nrow = n, ncol = p, dimnames = list(NULL, colnames(X)))

for (i in seq_len(n)) {
  cat("LOO sample:", i, "/", n, "¥n")

  model_i <- randomForest(
    x     = X[-i, , drop = FALSE],
    y     = y[-i],
    ntree = 1000
  )

  pred_loo[i] <- predict(model_i, X[i, , drop = FALSE], type = "prob")[, 2]

  shap_loo[i, ] <- fastshap::explain(
    object       = model_i,
    X            = X[-i, , drop = FALSE],
    newdata      = X[i, , drop = FALSE],
    pred_wrapper = function(m, newdata) predict(m, newdata, type = "prob")[, 2],
    nsim         = 1000
  )
}

shap_result <- data.frame(
  sample        = rep(rownames(X), p),
  feature       = rep(colnames(shap_loo), each = n),
  shap_value    = as.vector(shap_loo),
  feature_value = as.vector(as.matrix(X))
) %>%
  group_by(feature) %>%
  mutate(mean_abs_shap = mean(abs(shap_value))) %>%
```

```
ungroup()
```
