# Supplementary material for: Gut microbiome functional pathways outperform taxonomic profiles in predicting immune checkpoint inhibitor response in non-small cell lung cancer: an interpretable machine learning approach with SHAP
Source: Front Immunol. 2026 May 15;17:1832317. doi: 10.3389/fimmu.2026.1832317 (PMC13218901; doi:10.3389/fimmu.2026.1832317)
Supplement: Supplementary file 3 [file DataSheet3.pdf]

Supplementary Material

1 Supplementary Data

Table S1 (.csv file)

Analysis Code (.R file)

Figure S1

Figure S2

Figure S3

2 Supplementary Figures

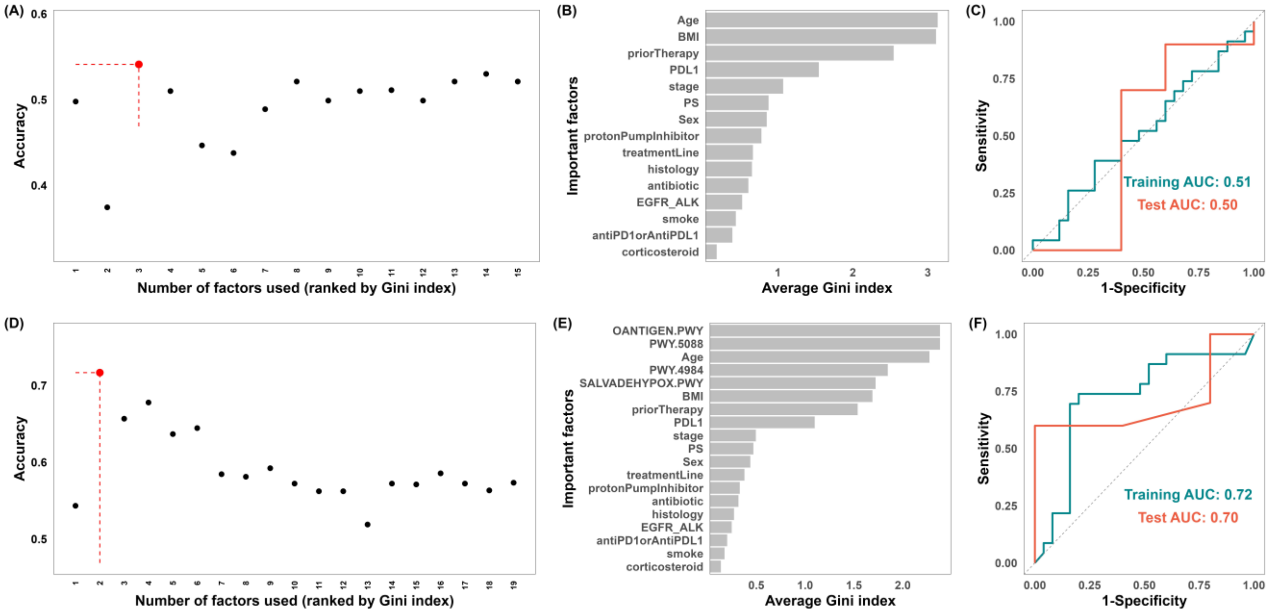

Figure S1. Predictive performance of models based on **(A)-(C)** clinical features alone and **(D)-(F)** combined clinical and microbial features (N = 63) for predicting RECIST-defined ICI response. **(A)** and **(D)**: The optimal feature subset was determined by incorporating the three and two most important features. **(B)** and **(E)**: Features ranked by Gini index. **(C)** and **(F)**: The corresponding ROC curves of training and test sets. Abbreviations: AUC, area under the receiver operating characteristic curve; ICI, immune checkpoint inhibitor; RECIST, Response Evaluation Criteria in Solid Tumors; ROC receiver operating characteristic curve.

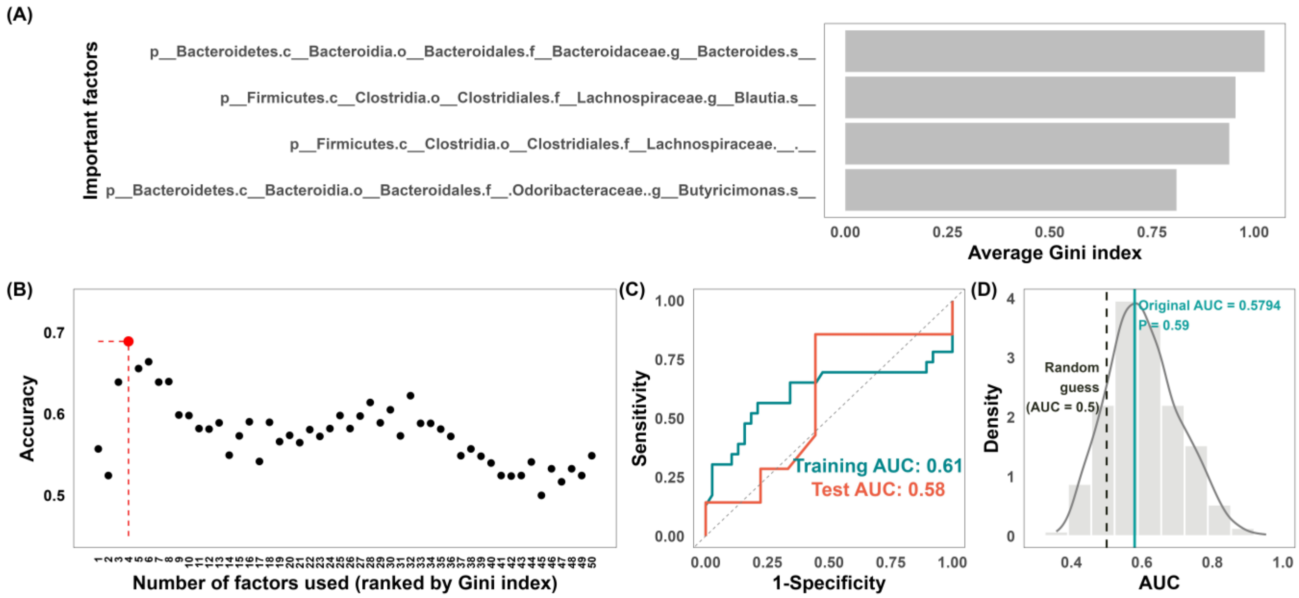

Figure S2. Predictive performance of model based on microbial taxonomic features (species) for predicting occurrence of irAEs (N=77). **(A)** The selected features ranked by Gini index. **(B)** The optimal feature subset was determined by incorporating the four most important features. **(C)** The corresponding ROC curves of training and test sets. **(D)** AUCs of permutation testing on the test set: times of permutation, 1,000. Abbreviations: AUC, area under the receiver operating characteristic curve; irAE, immune-related adverse events; P, P value; ROC receiver operating characteristic curve.

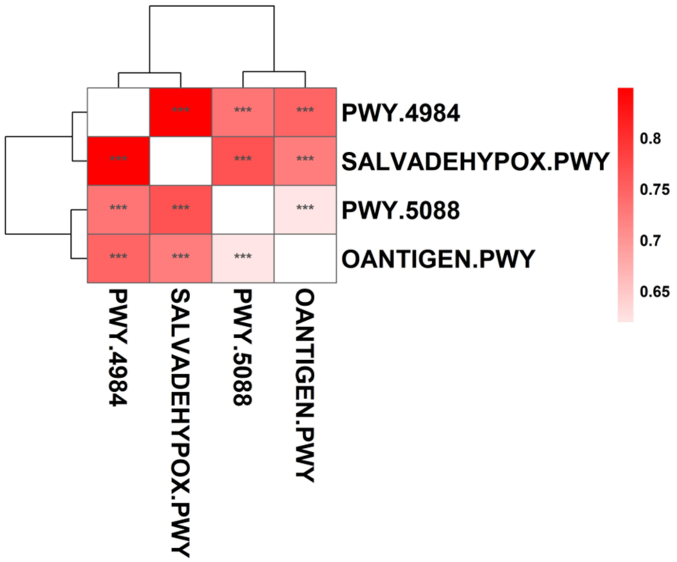

Figure S3. Spearman correlation matrix among the four pathways based on feature levels: PWY-4984 (urea cycle), SALVADEHYPOX-PWY (adenosine nucleotide degradation), OANTIGEN-PWY (O-antigen building blocks biosynthesis in *E. coli*), and PWY-5088 (L-glutamate degradation VIII to propanoate); *** P < 0.001. Abbreviations: P, P value.
